# *In Silico* Adoption of an Orphan Nuclear Receptor NR4A1

**DOI:** 10.1371/journal.pone.0135246

**Published:** 2015-08-13

**Authors:** Harald Lanig, Felix Reisen, David Whitley, Gisbert Schneider, Lee Banting, Timothy Clark

**Affiliations:** 1 Computer-Chemie-Centrum der Friedrich-Alexander-Universität Erlangen-Nürnberg, Nägelsbachstraße 25, 91052, Erlangen, Germany; 2 ETH Zürich, Institute of Pharmaceutical Sciences, Wolfgang-Pauli-Straße 10, 8093, Zürich, Switzerland; 3 Centre for Molecular Design, School of Pharmacy and Biomolecular Sciences, University of Portsmouth, King Henry Building, Portsmouth, PO1 2DY, United Kingdom; 4 School of Pharmacy and Biomolecular Sciences, University of Portsmouth, St. Michael’s Building, White Swan Road, Portsmouth, PO1 2DT, United Kingdom; Florida International University, UNITED STATES

## Abstract

A 4.1μs molecular dynamics simulation of the NR4A1 (hNur77) apo-protein has been undertaken and a previously undetected druggable pocket has become apparent that is located remotely from the ‘traditional’ nuclear receptor ligand-binding site. A NR4A1/bis-indole ligand complex at this novel site has been found to be stable over 1 μs of simulation and to result in an interesting conformational transmission to a remote loop that has the capacity to communicate with a NBRE within a RXR-α/NR4A1 heterodimer. Several features of the simulations undertaken indicate how NR4A1 can be affected by alternate-site modulators.

## Introduction

### Protein-ligand interactions and protein dynamics

“Orphan” nuclear receptors exhibit no obvious ligand-binding pocket in their X-ray crystal structures; many of their biological functions are poorly understood. They thus represent unique challenges for structural and mechanistic biology. We now describe a purely computational approach to this problem that takes advantage of recent developments both in our understanding of protein dynamics and in the technology of biological simulation.

The static “lock and key” view of protein-ligand binding has given way to a more dynamic interpretation known variously as the Monod–Wyman–Changeux (MWC, concerted or symmetry) model, [1–4] the two-state allosteric model, [5] the pre-equilibrium model, [1] or the “new view” of allosteric binding. [6–9] Cui and Karplus [8] have pointed out the importance of molecular-dynamics (MD) simulations in this picture. Indeed, apart from “slow” spectroscopic techniques such as NMR-spectroscopy and technically difficult trapping experiments, [9] MD simulations represent one of the very few tools able to provide a detailed description of protein dynamics in solution.

This dynamic view suggests that the accessible conformations of the protein can be characterized in MD simulations of a ligand-free protein provided (a) the force field used is accurate enough and (b) the time scale of the conformational inter-conversion is short enough to be accessible to MD simulations. Modern protein force fields, such as ff99SB [10] used here, represent years of development that have made them capable of reproducing biological conformations accurately and reliably. There is now ample indication in the literature that simulations of 100 ns and longer can reveal competing allosteric conformations. [11–15] A timely review of protein flexibility and function has recently appeared. [16]

Here we report an extensive MD and cheminformatics investigation of the NR4A1 receptor, an orphan receptor.

### NR4A1

The nuclear receptors (NRs) are a sizable class of transcription factors. Lipophilic ligands (steroids, fatty acids, retinoids and thyroid hormones, etc.) cause their translocation, normally from the cellular cytoplasm to the nucleus, where they initiate transcription of cognate genes. [17] NRs play essential roles in controlling cellular metabolism, division and proliferation in many tissues [18, 19] and are part of the steroid receptor superfamily. NR4A1 is often expressed in response to stress stimuli and cellular growth-factor signaling. NR4A1 and its subfamily appear to be constitutively active, modulated at the cellular level by differing ratios of their cognate NR coactivators and repressors and NR post-translational modification, including phosphorylation [20] and acetylation [21] but are currently classed as ‘orphan’ receptors. NR4A1 is expressed at low to moderate levels in many major physiological systems, including the central nervous system, endocrine, reproductive, immune, gastrointestinal, cardiovascular, respiratory and structural. [22, 23] It is known to play a vital role in tumor-cell apoptosis from multiple tissue types and in the well-studied apoptotic signaling of thymocytes [24] and within the hypothalmic-pituitary axis. [25]

The structural biology of NR4A1 is inconclusive. A crystal structure (PDB-ID [26] 2QW4) [27] of human NR4A1 reveals that the region of the “normal” ligand-binding domain is blocked by hydrophobic residues. [28] NMR studies have shown the closely related Nurr1 to undergo a conformational shift between “in” and “out” states, in which a comparable sequence of hydrophobic residues perform a “self-binding” function, [29] on the NMR time scale.

A hydrophobic co-regulator cleft comprised of helices 3, 5 and 12 is found in many members of the family. It plays a vital role in recruiting co-activators and co-repressors during gene transcription and its access is normally modulated by helix 12 within the non-orphan NRs, which undergo a conformational change on ligand binding. [30] However, this cleft is surprisingly hydrophilic in NR4A1, ruling out a similar role. Furthermore, partial denaturation studies suggest that NR4A1 has an unusually flexible helix 12.

**Fig 1:**
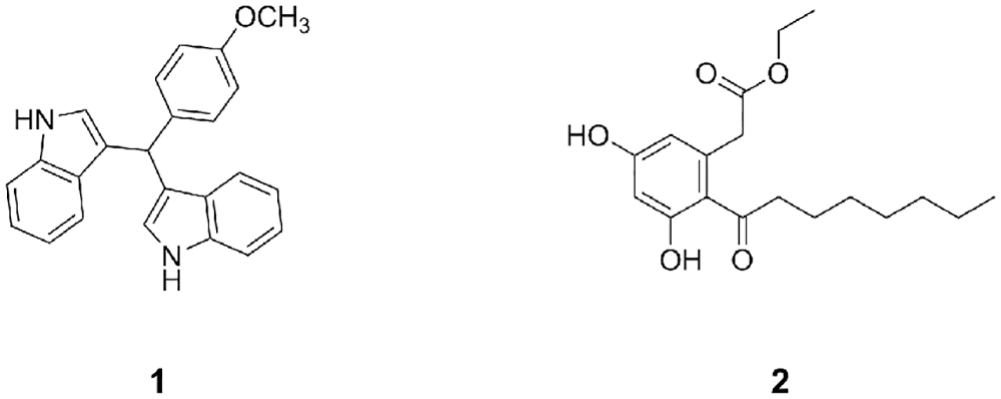
The structures of known small-molecule modulators of NR4A1.

Our interest was aroused by reports [31–33] of small-molecule modulators of NR4A1 action (**1** and **2**, see Fig 1). These may be viewed as Y-shaped hydrophobic ligands of the same general type that bind to ‘normal’ ligand-binding domains of other nuclear receptors.

Structurally different small molecule modulators of NR4A1 and Nurr1 are also known, [33] although their mode of binding and mechanism of action remain unknown. These observations suggest that NR4A1 exhibits druggable binding regions for which effective modulators can be developed analogously to those for the ERR receptors, which were originally thought not to have accessible ligand-binding domains. In this context, it is important that Katzenellenbogen *et al*. [34] have discussed Nuclear Receptor Alternate-Site Modulators (NRAMs), which bind to alternative pockets than the classical ligand-binding domain.

## Results and Discussion

Our purely calculational characterization of the structural and mechanistic biology of NR4A1 draws on a wide variety of simulation, analysis and bioinformatics techniques. Briefly, the questions that we have tried to answer are:

- Can we identify a binding pocket by MD simulations on the *apo*-receptor?
- If so, do the known modulators **1** and **2** bind to this pocket?
- Can we identify candidates for the native ligand(s)?
- What is the conformational effect of binding ligands on the receptor?
- What is the mode of their modulation?

### Conformations

The apo-NR4A1 simulation system consisted of the 233-residue ligand binding domain of NR4A1 reported (PDB-entry 2QW4, X-ray resolution: 2.8 Å). [27] Three amino acids (EPQ) that comprise the surface-loop region of a helix-turn-helix motif are not structurally resolved and were modeled into the structure. Details of the system and simulation setup are given in S1 Text and S2 Text. A single simulation of 4.1 μs was used to investigate the dynamics of the *apo*-protein.

**Fig 2:**
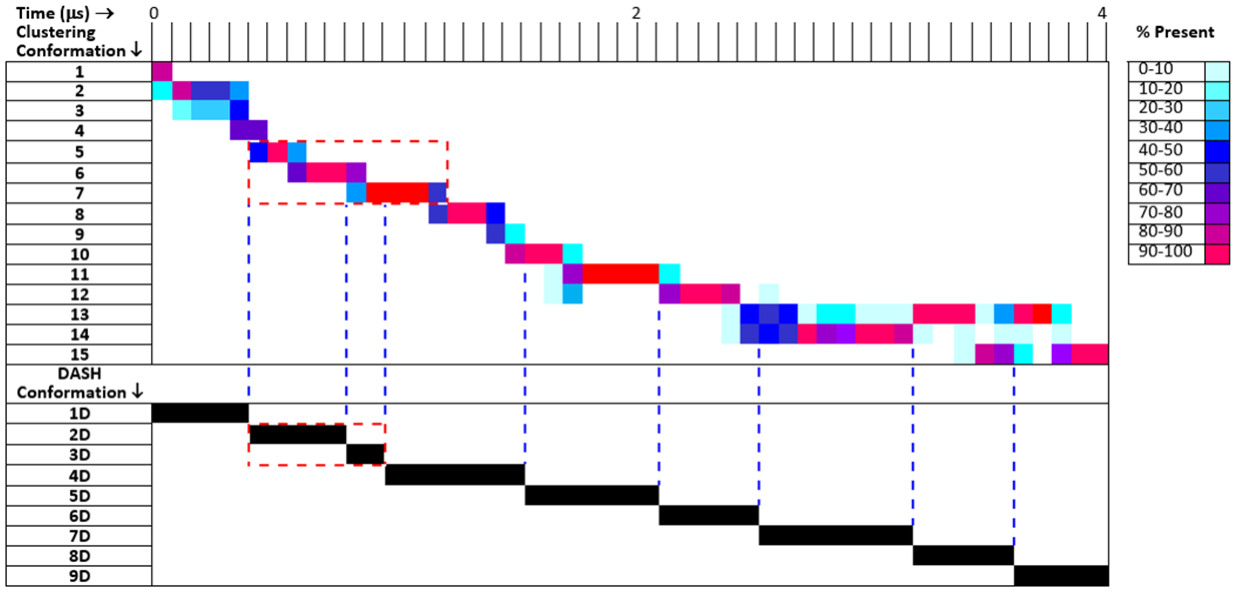
Clustering (above) and DASH, [35] (below) analyses of the 4.1 μs simulation of NR4A1. The clustering results are color-coded to indicate the population of the cluster in the given time period. The blue vertical dashed lines indicate transitions detected by DASH. The red dashed boxes indicate the clusters/conformations in which the binding pocket discussed below was found.

The conformations found in the resulting trajectory were identified and analyzed using both classical RMSD-based clustering and a DASH-analysis [35] based on 4,100 snapshots of the backbone dihedral angles taken every nanosecond (see S3 Text for details). The results of the two analyses are compared (see Fig 2). Transitions between conformations identified by DASH correspond well to those indicated by the cluster analysis. DASH conformation 3D lies between clustering conformations 6 and 7 and cluster conformations 13, 14 and 15, which switch frequently in the last μs of the simulation; these are interpreted by DASH as three sequential conformations (7D, 8D and 9D). The clustering analysis reveals a series of essentially irreversible (in the context of this simulation) conformation changes between 15 conformations that eventually results in equilibrium between conformations 12–14 after approximately 3.5 μs. The X-ray conformation itself, which corresponds to conformation 1 and 1D, is short-lived (although it would be considered stable by accepted MD standards as it is observed for the first 83 ns of the simulation).

The apparently irreversible conformational change observed during the simulation is a movement of the loop near the N-terminus (residues 175–185) from an “open” to a “closed” conformation by approaching the N-terminal end of helix 1 (see S4 Text). This motion closes a “novel” binding pocket discussed below. A second important feature of the simulation is that helix 1 unfolds and refolds repeatedly.

### Pocket analysis

**Fig 3:**
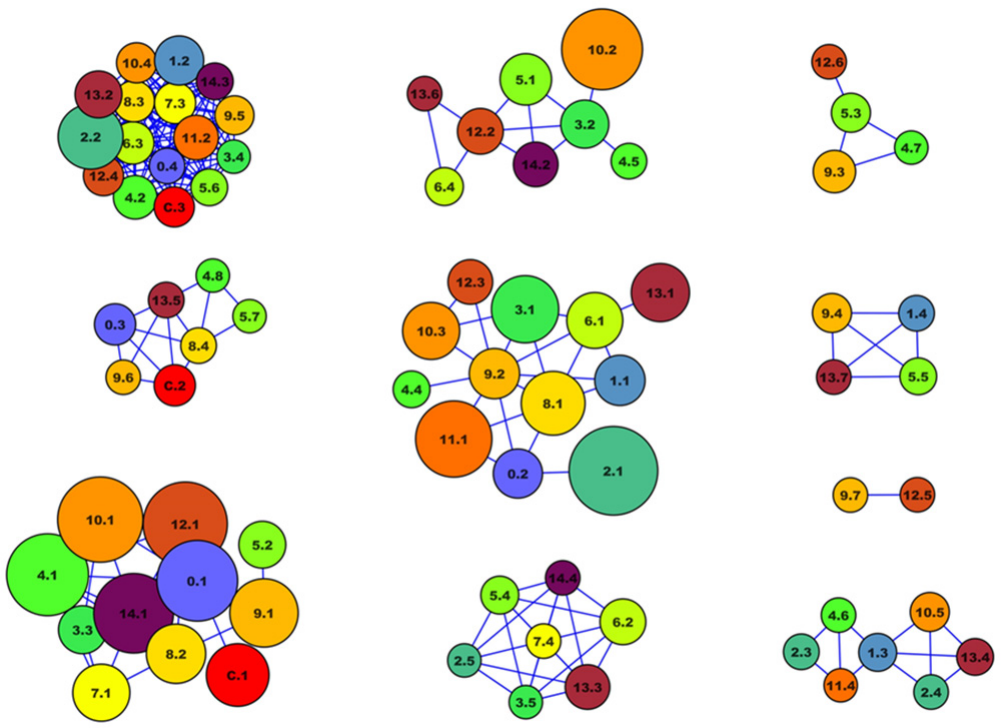
Clustering of pockets that occur in the crystal structure and different snapshots of the MD simulation. Each vertex represents one pocket. Vertices are connected if the pockets are located in similar regions of the protein surface and have a mutual overlap of at least 30%. Vertex labels consist of the cluster number (0–14, c = crystal) and the pocket number (as identified by PocketPicker,^36^ sorted by size in ascending order). Vertex colors and sizes correspond to the number of the snapshots (red: crystal) and the pocket sizes, respectively. Clusters **1**–**3** contain pockets that are present both in the crystal structure and in some snapshots. Pockets of clusters **4–10** are not present or smaller than 100 Å^3^ in the crystal structure. Cluster **4** (light green) represents a potential binding site. Clusters **5–10** represent pockets whose shapes or sizes render ligand binding unlikely.

A PocketPicker [36] analysis was conducted for the 15 cluster-centers found by the classical clustering of the 4,100 MD snapshots (the default settings described in reference [36] were used). Large (≥ 100 Å^3^) pockets that are apparent in multiple cluster representatives but not in the crystal structure were extracted for each structure, their pairwise overlaps calculated, and the results organized in graphs (see Fig 3). Each vertex represents one pocket. The vertex size is proportional to the volume of the pocket and the color encodes the MD-cluster number. Vertices are connected if the pockets overlap by at least 30%. Vertices without emerging edges are not shown. Highly connected clusters of large vertices indicate potential binding sites that are not present in the crystal structure if they do not contain vertices that represent pockets of the crystal.

**Fig 4:**
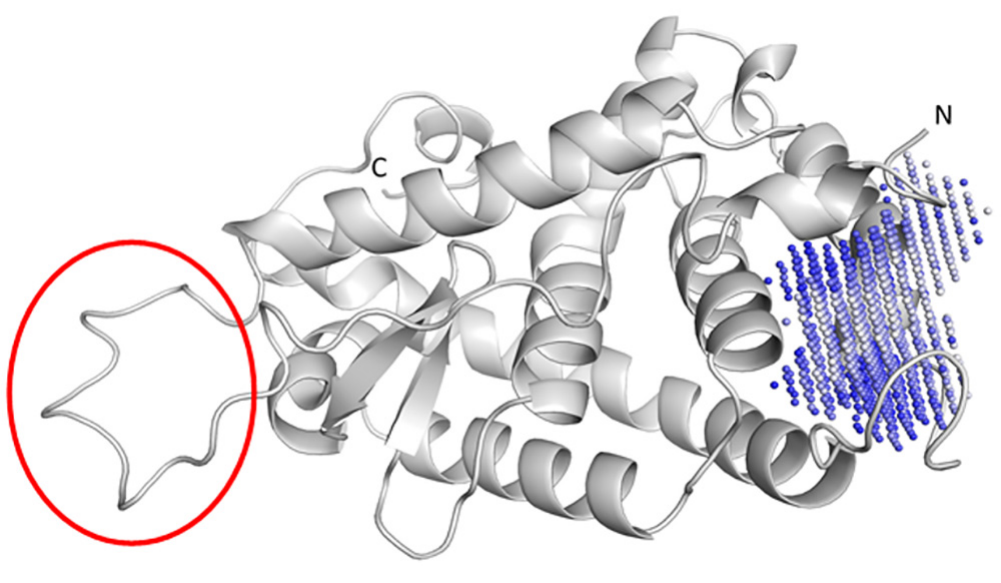
The predicted binding site (blue/blue grey spheres) is located close to the N-terminus of NR4A1. The flexible loop that is stabilized by ligand binding is indicated by the red ellipse.

Visual inspection reveals that a potential binding pocket, present in the snapshots indicated (see Fig 3), is flanked by the residues Leu6, Pro139, Cys172, Pro184; this pocket is shown in Fig 4. This clear druggable pocket is found in clusters 5, 6 and 7 and in DASH conformations 2D and 3D and thus exists continuously for 500–800 nanoseconds of the simulation before the N-terminal flap closes.

This pocket remained stable for at least 1 μs in an independent MD simulation starting with the empty pocket found in the cluster analyses. A slight decrease in the pocket volume caused by the flexibility of the surface loop around Val180 is found, but the pocket is still present.

**Fig 5:**
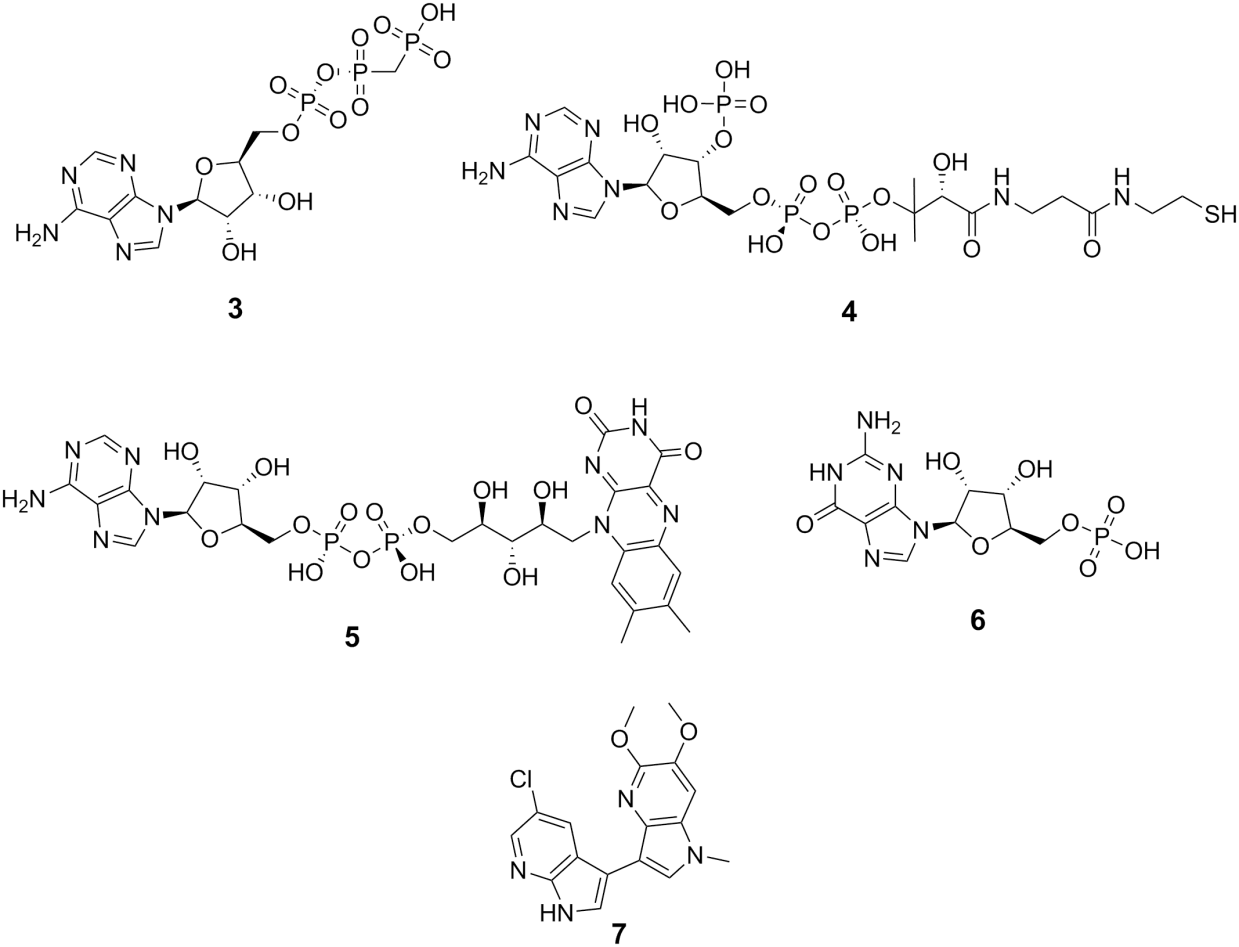
Putative ligands for the newly found binding site in NR4A1.

A PoLiMorph [37] pocket graph description was then used to search for pockets structurally related to this potential binding site in the scPDB. [38] Assuming that similar binding sites bind to similar ligands, we extracted all bound ligands from the crystal structures of the most similar binding sites (PoLiMorph *score* ≥ 0.15 using the default settings described in reference [35]). Four of these nine ligands (shown in Fig 5) are nucleotides; adenosine-5′-β,γ-methylene triphosphate (**3**), coenzyme A (**4**), FAD (**5**) and guanosine-5′-monophosphate (**6**). We also found a *bis*-aza-indole compound **7** among the top ranked molecules, which is remarkable since one of the known NR4A1 activators is *bis*-indole **1**.

### Binding Known Ligands

Ligands **1** and **2** could both be docked well into the new pocket (for details see S5 Text) using Autodock4. [39] The best docked poses were then used as starting points for 1 μs MD simulations to assess the stability of the ligands in the pocket and their effect on the conformation of the protein.

**Fig 6:**
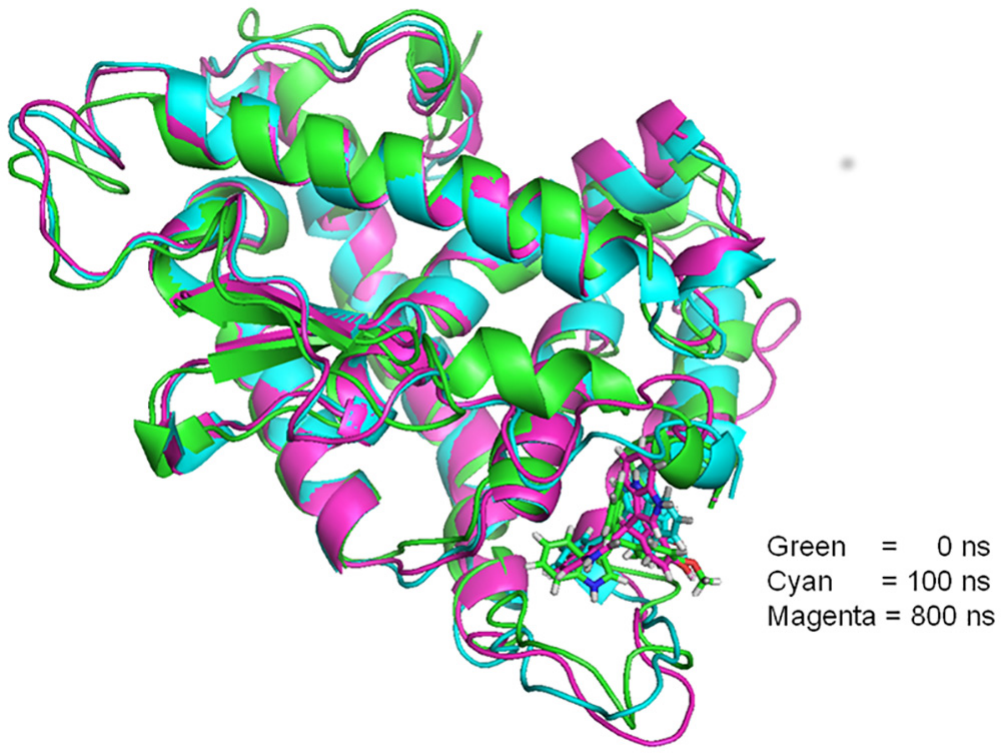
Representative snapshots taken from the MD simulation of ligand 1 within the new binding pocket (cyan, after 100 ns; magenta, after 800ns), fitted onto the starting geometry of the docked complex (green).

RMSD plots of the protein α-carbon atoms and all ligand atoms (see S6 Text) show that the protein conformation remains stable over the 1 μs simulation, with the C_α_-RMSD from the starting geometry varying between 2.5 and 3.5 Å after an initial 100 ns relaxation period. The ligand-RMSD plot also shows an essentially stable 3 Å RMSD with frequent excursions to structures with 7–8 Å RMSD from the starting structure. For ligand **1**, closer examination shows that the structure with 3 Å RMSD, which corresponds to the 100 ns snapshot shown (see Fig 6b), involves movement of the ligand deeper into the pocket than is the case in the docked structure (see Fig 6a).

The second conformation found periodically in the simulation with increasing frequency after 200 ns corresponds to the 800 ns structure shown in Fig 6. In this structure, helix 1, which unravels and reforms repeatedly during the last half of the simulation, is unraveled and the ligand takes up a position deep in the pocket, but shifted towards the original position of helix 1. Thus, the simulation indicates that ligand **1** indeed binds tightly to the newly found pocket.

The RMSD plot of the α-carbon atoms for the simulation starting with docked ligand **2** also shows that the protein maintains its secondary and tertiary structure for the whole 1 μs simulation. The only exception is helix 1, which is again affected by the presence of the ligand, which causes the helix to unwind and rewind repeatedly. For this reason, the highly flexible ligand is able to move and reorient within the binding pocket, as can be shown by an overlay of representative snapshots extracted from the trajectory (see S7 Text). The flexible alkyl chains of ligand **2** explore the dynamically changing binding cleft more effectively than observed for compound **1**. This is supported by the considerably higher ligand RMSD values (8–9 Å). Nevertheless, ligand **2** remains firmly in the binding pocket defined by the flexible loop 175–185.

Starting at 780 ns, the loop forming the new binding pocket shifts towards helix 1 and closes the pocket, so that the ligand is completely trapped. This closing is only possible because the ligand adopts a position above helices 8 and 9, flanked by the C-terminus of helix 2, additionally covered by the very flexible N-terminus of the protein. The highly flexible side chains of compound **2** intercalate between the helix bundles of the protein, making binding even tighter.

As a further check of the relevance of the newly found binding site, we docked five known [40–42] active NR4A1 bisindole ligands into this site (details of the docking runs and the results are given in S5 Text). All five give good binding poses with high scores and adopt the same binding mode as found for **1** and **2** above.

### Effects of Ligand Binding

What are the consequences of the above results in identifying a biological role and mechanism for NR4A1? Ligand binding at the newly identified site induces conformational changes both adjacent to the pocket and in the remote flexible loop area highlighted in Fig 4. The more remarkable of these two effects is a conformationally transmitted stabilization of the flexible loop (^25^FQELVLPHFGKEDAGD^40^), even though it is quite remote from the binding site.

**Fig 7:**
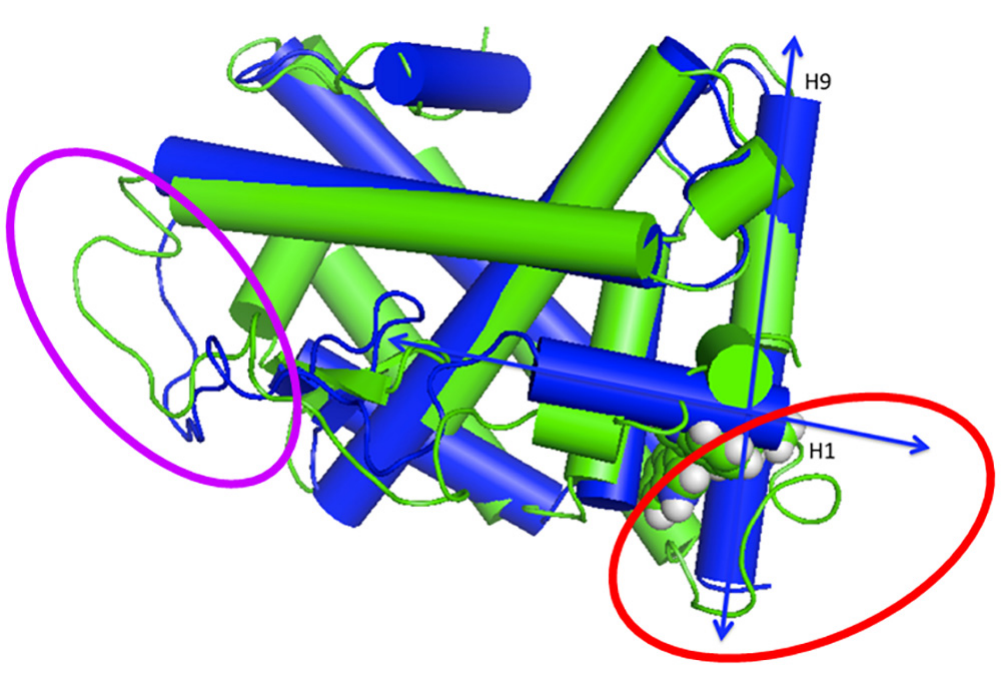
Apo-NR4A1 (blue, PDB:2QW4) compared to a snapshot of NR4A1 after 45ns simulation in the presence of 1 (green). The blue double headed arrows indicate the axes of the helices 1 and 9 in the apo-structure. The regions of the protein that rearrange on ligand binding are denoted by the ellipses (Red; adjacent to the binding site. Violet; remote loop ^25^F FQELVLPHFGKEDAGD-D^40^).

The pocket itself is close to the ‘hinge’ region of the ligand-binding domain. As outlined above, helix 1 becomes very labile on ligand binding. Fig 7 shows that helix 9 is also partially unraveled (color-coded green) compared to the apo-structure (color-coded blue).

The newly formed loop region adjacent to the binding site (^173^LKEHVAAVAGEPQPAS^188^, red ellipse in Fig 7) is close to a to a well-recognized hinge region between the ligand- and DNA-binding domains, as indicated by a modeled alignment with the PPAR-γ receptor bound to a cognate Peroxisome Proliferator Response Element (PPRE) [43] (see S8 Text). This comparison is equivalent to the known hetero-dimerization of NR4A1 with RXR-α. [44] Changes in this hinge region can result in alterations in signaling because the hinge region of NRs can contain elements of the nuclear localization signal. [45] NR4A1 has a predicted [46] nuclear localization signal (^358^RRGRLPS^365^K) upstream of our modeled protein extremely close to the N-terminus of the predicted ligand-binding domain. Thus, changes adjacent to the newly identified binding site may switch NR4A1 localization, which in turn is intimately correlated to its apoptotic function. [47] An identified nuclear export sequence under the control of MEK-ERK-RSK cascade also lies close to the cognate nuclear localization sequence, with phosphorylation being effected by RSK. [48] This region also attracts the attention of an additional serine kinase MSK that operates in fibroblast cellular stress pathways. [49] Structural changes in this vital region are highly likely to affect subcellular location of NR4A1. [50]

**Fig 8:**
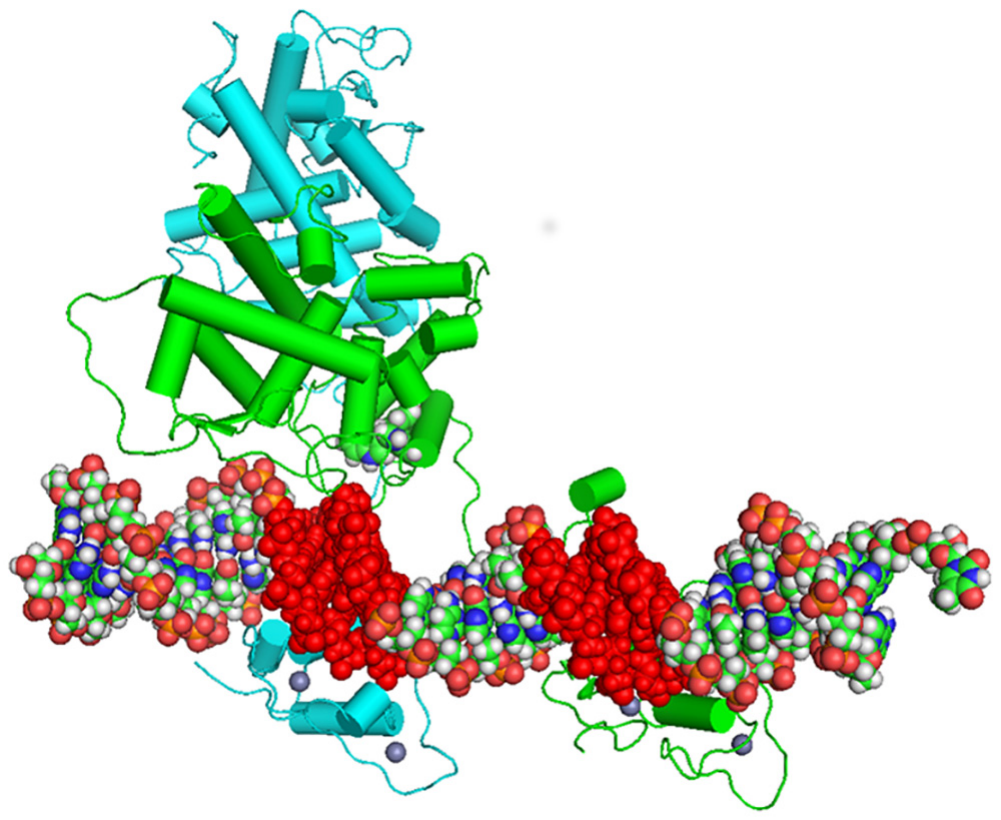
Model of the apo-NR4A1 (green cylinders) in dimerization with apo-RXR-α (blue cylinders) at the NBS of a cognate NBRE, DNA (CPK colors) indicates the NBS sequences (Red) and the location of the *bis*-indole 1 (CPK spheres) post-superimposition of the NR4A1 45ns complex.

A less direct, but fascinating possibility is that the allosteric rearrangement observed for the remote loop ^25^FQELVLPHFGKEDAGD^40^ (the violet ellipse in Fig 7 and violet loop in Fig 8) is involved in biological regulation by modifying heterodimer stability and binding at a cognate NuRE. Fig 8 shows a hypothetical alignment of a response element with a heterodimer of a *bis*-indole complexed NR4A1 and RXR-α based on the PPRE PPAR-γ and RXR-α X-ray structure [43] and informed by SAXS data for the VDR/RXR-α dimer at its response element. The region of NR4A1 highlighted by our simulations to change (labeled blue A) on ligand binding is close to a similar loop region in the RXR monomer (labeled red B). This suggests that protein/protein communication between heterodimer partners in this region of the complex can lead to modulation of DNA binding. It is known that rexinoid AHPN has influence on both the RXR/NR4A1 dimer’s transcription processes and on localization because the dimer is transported from the nucleus to the mitochondria. [50]

### Construction of a NR4A1 / RXR heterodimeric complex model located on a NBS at a cognate NRBE

In an attempt to understand more fully the effect of the allosteric binding of the NR4A1 *bis*-indole ligand, a model of a NR4A1/RXR heterodimer at a NBS (hNurr77 binding sequence) on a recognized NBRE, the RAR β2 [51] was constructed. The RAR β2 sequence was obtained [52] and modelled [53] to obtain ‘raw’ structural coordinates. The coordinates of the DBDs of RXR-α and NR4A1 were obtained (PDB: 3DZY, 1CIT respectively) and positioned with their preferred ‘polarity’ at their cognate binding sequences by a process of coordinate mapping *via* the MacPymol *align* routine working with the crystallographic base pair coordinates of the respective DBD/DNA complexes onto a modelled RARβ2 followed by editing. The monomer coordinates of the LBDs of RXR-α and NR4A1 (PDB: 3DZY, 2QW4) were mapped onto those of the PPAR/RXR-α heterodimer crystallographic coordinates (PDB: 3DZY) in the absence of the linking sequences that bridge the respective LBDs and DBDs. In doing so there is an implicit assumption that the dimer interface of the modelled complex adopts the orientation of the PPAR/RXR-α heterodimer. The modelled heterodimer was posed, using the translational tools within MacPymol, approximately 23 Å above the RARβ2 (roughly equidistant from each DBDs). The linking sequences were modelled using the loop-building functionality of Swiss-PdbViewer, followed by energy minimization of the new protein fragments. The final model was checked for sequence and backbone dihedral angle consistency.

Following an 85.2 ns MD simulation the resultant complex showed a considerably different configuration than its starting point. It was pleasing to see that, during the course of the simulation, the complex had reoriented to adopt a configuration reminiscent of that observed (SAXS/SANS/FRET) for the solution structure of the RXR/RAR heterodimer at a DR5 response element with the RXR binding at the 5’ and the NR4A1 at the 3’ half sites respectively, [54, 55] (see Fig 8).

The *bis*-indole/NR4A1 modelled complex coordinates were posed against the resultant coordinates of the NR4A1 component of the heterodimer complex at the NBRE using MacPymol *align*. This pose highlights the close proximity of the bis-indole to a critical ‘hinge’ region of the NR4A1 complex (see Fig 8) and to the DNA binding interface, the detail of which will be the subject of further study.

The key reviews, [33,40] covering the targeting of NR4A1 for cancer treatment, reveal a high degree of complexity in the potential modes of action of **1**, and its analogues, which appear to act differently on varying tumor types and *via* NR4A1 partner protein complexes. It is suggested that the *p*-hydroxy analogue of **1** acts on the A/B domain of NR4A1 whereas **1** on the LBD within the E/F domain, fluorescence studies [56] confirm this. X-ray crystallography of the NR4A1 homodimeric LBD show that small molecule Cytosporone B-like ligands of NR4A1 such as ethyl 2-[2,3,4-trimethoxy-6-(1-octanoyl)phenyl]acetate (TMPA) are also able to bind promiscuously. [32, 57–58] Taken together, this evidence suggests strongly that NR4A1 may be modulated by small molecules in a non-classical way, at multiple sites, for a member of the steroid receptor superfamily.

What is emerging is that allosteric modulation of the NRs is evident and that these approaches have predictive potential when considering ‘transcriptional machinery’ stability [59, 60] and that this aspect may be influenced by small molecules.

Although many of the biological functions of NR4A1 are still unclear, the simulations have provided answers to the questions posed in the introduction and have revealed fascinating features that point to new research directions. The simulations reveal a pronounced pocket that binds the known ligands and is similar to known nucleotide-binding sites. Binding ligands in this pocket induces two changes; a new loop adjacent to the binding site that is well placed to switch DNA binding and a stable loop in a remote region of the protein that has the capacity to affect localization at the NBRE. These are remarkable conclusions for a protein that has resisted characterization experimentally. MD simulations combined with detailed analyses of the trajectories have proven to be a powerful tool for exploratory research of protein structure and function.

## Supporting Information

S1 Structures pdb files of the structures discussed in the manuscript and Supporting Information. (ZIP)

S1 Text Structure generation. (PDF)

S2 Text MD simulations. (PDF)

S3 Text Clustering and DASH analyses. (PDF)

S4 Text Structures of the clusters and N-terminal loop-closing. (PDF)

S5 Text Docking of known ligands into the new pocket. (PDF)

S6 Text RMSD data ligand 1 complex. (PDF)

S7 Text Details of ligand 2 binding simulations. (PDF)

S8 Text A RXR-α / hNur77 apo-dimer at a cognate NBRE. (PDF)

## References

[1] Changeux J-P. Allosteric interactions interpreted in terms of quaternary structure. Brookhaven Symposia in Biology, 1964;17: 232–249. 14246265

[2] Monod J, Wyman J, Changeux J-P. On the nature of allosteric transitions: a plausible model. J. Mol. Biol. 1965;12: 88–118. 14343300 10.1016/s0022-2836(65)80285-6

[3] Changeux J-P, Edelstein SJ. Allosteric receptors after 30 years. Neuron 1998;21: 959–980. 10.1016/S0896-6273(00)80616-9 9856454

[4] Changeux J-P, Edelstein SJ. Allosteric mechanisms of signal transduction. Science, 2005; 308: 1424–1428. 10.1126/science.1108595 15933191

[5] Hall DA. Modeling the functional effects of allosteric modulators at pharmacological receptors: an extension of the two-state model of receptor activation, Mol. Pharmacol. 2000;58: 1412–1423. 10.1124/mol.58.6.1412 11093781

[6] Kale S, Jordan F. Conformational ensemble modulates co-operativity in the rate-determining catalytic step in the E1 component of the *Escherichia coli* pyruvate dehydrogenase multienzyme complex, J. Biol. Chem., 2009;284: 33122–33129. 10.1074/jbc.M109.065508 19801660 PMC2785154

[7] Gunasekaran K, Ma B, Nussinov R. Is allostery an intrinsic property of all dynamic proteins. Proteins, 2004;57: 433–443. 10.1002/prot.20232 15382234

[8] Cui Q, Karplus M. Allostery and co-operativity revisited, Protein Sci., 2008;17: 1295–1307. 10.1110/ps.03259908 18560010 PMC2492820

[9] Viappiani C, Bettati S, Bruno S, Ronda L, Abbruzzetti S, Mozzarelli A, et al New insights into allosteric mechanisms from trapping unstable protein conformations in silica gels, Proc. Natl. Acad. Sci. USA, 2004;101: 14414–14419. 10.1073/pnas.0405987101 15385676 PMC521967

[10] Hornak V, Abel R, Okur A, Strockbine B, Roitberg A, Simmerling C. Comparison of multiple Amber force fields and development of improved protein backbone parameters, Proteins, 2006;65: 712–725. 10.1002/prot.21123 16981200 PMC4805110

[11] Dror RO, Arlow DH, Borhani DW, Jensen MØ, Piana S, Shaw DE. Identification of two distinct inactive conformations of the β2-adrenergic receptor reconciles structural and biochemical observations, Proc. Natl. Acad. Sci. USA, 2009;12: 4689–4694. 10.1073/pnas.0811065106 PMC2650503 19258456

[12] Vanni S, Neri M, Tavernelli I, Röthlisberger U. Predicting novel binding modes of agonists to β adrenergic receptors using all-atom molecular dynamics simulations., PLoS Comput. Biol. 2011;7: e1001053, 10.1371/journal.pcbi.1001053 21253557 PMC3017103

[13] Khalili-Araghi F, Gumbart J, Wen PC, Sotomayor M, Tajkhorshid E, Schulten K. Molecular dynamics simulations of membrane channels and transporters. Curr. Opin. Struct. Biol. 2009;19: 128–137. 10.1016/j.sbi.2009.02.011 19345092 PMC2680122

[14] Haberl F, Lanig H, Clark T. Induction of the tetracycline repressor: characterization by molecular-dynamics simulations. Proteins Struct. Funct. Bioinf., 2009;77: 857–866. 10.1002/prot.22505 19626707

[15] Tanrikulu Y, Proschak E, Werner T, Geppert T, Todoroff N, Klenner A, et al Homology-model adjustment and ligand screening with a pseudo-receptor of human histamine H4 receptor. ChemMedChem, 2009;4: 820–827. 10.1002/cmdc.200800443 19343764

[16] Gross M. Anarchy in the proteome. Chemistry World, 2011;8: 42–45.

[17] Maxwell MA, Muscat GE. The NR4A subgroup: immediate early response genes with pleiotropic physiological roles, Nucl. Recept. Signal., 2006;4: e002 10.1621/nrs.04002 16604165 PMC1402209

[18] Pearen MA, Muscat GE. Nuclear hormone receptor 4A signaling: implications for metabolic disease. Mol. Endocrinol. 2010;24: 1891–1903. 10.1210/me.2010-0015 20392876 PMC5417389

[19] Berriel Diaz M, Lemke U, Herzig S. Discovering orphans' sweet secret: NR4A receptors and hepatic glucose production. Cell Metab., 2006;4: 339–340. 10.1016/j.cmet.2006.10.005 17084708

[20] Thompson J, Burger ML, Whang H, Winoto A. Protein kinase C regulates mitochondrial targeting of Nur77 and its family member Nor-1 in thymocytes undergoing apoptosis. Eur. J. Immunol., 2010;40: 2041–2049. 10.1002/eji.200940231 20411565 PMC3076209

[21] Wang C, Powell M, Tian L, Pestell RG. Analysis of nuclear receptor acetylation. Methods Mol. Biol., 2011;776: 169–181. 10.1007/978-1-61779-243-4_11 21796527

[22] Huang P, Vikas CV, Rastinejad F. Structural overview of the nuclear receptor superfamily: Insights into physiology and therapeutics. Ann. Rev. Physiol., 2010;72: 247–272. 10.1146/annurev-physiol-021909-135917 20148675 PMC3677810

[23] Hsu HC, Zhou T, Mountz JD. Nur77 family of nuclear hormone receptors. Curr. Drug Targets Inflamm. Allergy, 2004;3: 413–423. 10.2174/1568010042634523 15584889

[24] Winoto A, Littman DR. Nuclear hormone receptors in T lymphocytes. Cell, 2002;109: S57–S66. 10.1016/S0092-8674(02)00710-9 11983153

[25] Okabe T, Takayanagi R, Adachi M, Imasaki K, Nawata H. Nur77, a member of the steroid receptor superfamily, antagonizes negative feedback of ACTH synthesis and secretion by glucocorticoid in pituitary corticotrope cells. J. Endocrinol., 1998;156: 169–175. 10.1677/joe.0.1560169 9496246

[26] The RCSB Protein Data Bank. Available: http://www.uniprot.org/uniprot/P22736. Accessed 2015 April 29

[27] Min JR, Schuetz A, Loppnau P, Weigelt J, Sundstrom M, Arrowsmith CH, et al. Available: http://www.pdb.org/pdb/explore/explore.do?structureId=2QW4, Accessed 2015 April 29

[28] Flaig R, Greschik H, Peluso-Iltis C, Moras D. Structural basis for the cell-specific activities of the NGFI-B and the Nurr1 ligand-binding domain. J. Biol. Chem. 2005;280: 19250–19258. 10.1074/jbc.M413175200 15716272

[29] Michiels P, Atkins K, Ludwig C, Whittaker S, van Dongen M, Günther U. Assignment of the orphan nuclear receptor Nurr1 by NMR. Biomol. NMR Assign., 2010;4: 101–105. 10.1007/s12104-010-9210-4 20300892

[30] Wansa KD, Harris JM, Muscat GE. The activation function-1 domain of Nur77/NR4A1 mediates trans-activation, cell specificity, and co-activator recruitment. J. Biol. Chem., 2002;277: 33001–33011. 12082103 10.1074/jbc.M203572200

[31] Chinthalapally S, Burkhardt RD, Papineni S, Ramaiah S, Yoon K, Safe S. Activation of Nur77 by Selected 1,1-Bis(3’-indolyl)-1-(*p*-substituted phenyl)methanes Induces Apoptosis through Nuclear Pathways., J. Biol. Chem. 2005;280: 24903–24914. 10.1074/jbc.M500107200 15871945

[32] Zhan Y, Du X, Chen H, Liu J, Zhao B, Huang D, et al Cytosporone B is an agonist for nuclear orphan receptor Nur77, Nature Chem. Biol. 2008;4: 548–556. 10.1038/nchembio.106 18690216

[33] Lee SO, Li X, Khan S, Safe S. Targeting NR4A1 (TR3) in cancer cells and tumors., Expert Opin. Ther. Targets., 2011;15: 195–206. 10.1517/14728222.2011.547481 21204731 PMC4407471

[34] Moore TW, Mayne CG, Katzenellenbogen JA. Not picking pockets: nuclear receptor alternate-site modulators (NRAMs)., Mol. Endocrinol., 2010;24: 683–695. 10.1210/me.2009-0362 19933380 PMC2852352

[35] Salt DW, Hudson BD, Banting L, Ellis MJ, Ford MG. DASH: a novel analysis method for molecular dynamics simulation data. Analysis of ligands of PPAR-gamma, J. Med. Chem., 2005;48: 3214–3220. 10.1021/jm049216s 15857127

[36] Weisel M, Proschak E, Schneider G. PocketPicker: analysis of ligand binding-sites with shape descriptors. Chem. Central J. 2007;1: 7 10.1186/1752-153X-1-7 PMC1994066 17880740

[37] Reisen F, Weisel M, Kriegl JM, Schneider G. Self-Organizing Fuzzy Graphs for Structure-Based Comparison of Protein Pockets, J Proteome Res 2010;9: 6498–6510. 10.1021/pr100719n 20883038

[38] Kellenberger E, Muller P, Schalon C, Bret G, Foata N, Rognan D. sc-PDB: an annotated database of druggable binding sites from the Protein Data Bank, J Chem Inf Model 2006;46: 717–727. 10.1021/ci050372x 16563002

[39] Morris GM, Huey R, Lindstrom W, Sanner MF, Belew RK, Goodsell DS, et al AutoDock4 and AutoDockTools4: Automated docking with selective receptor flexibility, J. Comput. Chem., 2009;30: 2785–2791. 10.1002/jcc.21256 19399780 PMC2760638

[40] Lee SO, Li X, Hedrick E, Jin UH, Tjalkens RB, Backos DS, et al Diindolylmethane analogs bind NR4A1 and are NR4A1 antagonists in colon cancer cells, Mol. Endocrinol., 2014;28: 1729–1739. 10.1210/me.2014-1102 25099012 PMC4179635

[41] Lee SO, Jin UH, Kang JH, Kim SB, Guthrie AS, Sreevalsan S, et al The orphan nuclear receptor NR4A1 (Nur77) regulates oxidative and endoplasmic reticulum stress in pancreatic cancer cells, Mol. Cancer Res., 2014; 527–538. 10.1158/1541-7786.MCR-13-0567 24515801 PMC4407472

[42] Lee SO, Andey T, Jin UH, Kim K, Sachdeva M, Safe S. The nuclear receptor TR3 regulates mTORC1 signaling in lung cancer cells expressing wild-type p53, Oncogene, 2012;31: 3265–3276. 10.1038/onc.2011.504 22081070 PMC3299891

[43] Chandra V, Huang P, Hamuro Y, Raghuram S, Wang Y, Burris TP, et al Structure of the intact PPAR-gamma-RXR- nuclear receptor complex on DNA. *Nature*, 2008;456: 350–356. 10.1038/nature07413 19043829 PMC2743566

[44] Zetterström RH, Solomin L, Mitsiadis T, Olson L, Perlmann T. Retinoid X receptor heterodimerization and developmental expression distinguish the orphan nuclear receptors NGFI-B, Nurr1, and Nor1. Mol. Endocrinol., 1996;10: 1656–1666. 8961274 10.1210/mend.10.12.8961274

[45] Saporita AJ, Zhang Q, Navai N, Dincer Z, Hahn J, Cai X, et al Identification and characterization of a ligand-regulated nuclear export signal in androgen receptor. J. Biol. Chem., 2003; 278: 41998–42005. 10.1074/jbc.M302460200 12923188

[46] Nguyen Ba AN, Pogoutse A, Provart N, Moses AM. NLStradamus: A simple Hidden Markov Model for nuclear localization signal prediction. BMC Bioinformatics, 2009; 10: 202 10.1186/1471-2105-10-202 19563654 PMC2711084

[47] Brenner C, Kroemer G. Apoptosis. Mitochondria-the death signal integrators, Science, 2000;18; 289: 1150–1151. 10970229 10.1126/science.289.5482.1150

[48] Wang A, Rud J, Olson CM Jr, Anguita J, Osborne BA. Phosphorylation of Nur77 by the MEK-ERK-RSK cascade induces mitochondrial translocation and apoptosis in T cells, J. Immunol., 2009; 183: 3268–3277. 10.4049/jimmunol.0900894 19675165

[49] Darragh J, Soloaga A, Beardmore VA, Wingate AD, Wiggin GR, Peggie M, et al MSKs are required for the transcription of the nuclear orphan receptors Nur77, Nurr1 and Nor1 downstream of MAPK signaling, Biochem. J., 2005; 390: 749–759. 10.1042/BJ20050196 15910281 PMC1199668

[50] Zhang XK. Targeting Nur77 translocation, Expert Opin. Ther. Targets, 2007;11: 69–79. 10.1517/14728222.11.1.69 17150035

[51] Lefebvre P, Mouchon A, Lefebvre B, Formstecher P. Binding of retinoic acid receptor heterodimers to DNA. A role for histones NH_2_ termini, J. Biol. Chem., 1998;273: 12288–12295. 9575180 10.1074/jbc.273.20.12288

[52] From the Human genome (GRCh37, Gene: RARB ENSG00000077092 Chromosome 3, RARbeta 2 promoter, Release 72 –June 2013). Available: http://www.ensembl.org/Gene/Summary?g=ENSG00000077092. Accessed 2015 April 13

[53] van Dijk M, Bonvin AMJJ. 3D-DART: a DNA structure modelling server, Nucl. Acids Res., 2009;37 (Web Server Issue), W235–W239. 10.1093/nar/gkp287 19417072 PMC2703913

[54] Brélivet Y, Rochel N, Moras D. Structural analysis of nuclear receptors: from isolated domains to integral proteins, Mol. Cell. Endocrinol., 2012;348: 466–473. 10.1016/j.mce.2011.08.015 21888944

[55] Rochel N, Ciesielski F, Godet J, Moman E, Roessle M, Peluso-Iltis C, et al Common architecture of nuclear receptor heterodimers on DNA direct repeat elements with different spacings. Nat. Struct. Mol. Biol., 2011;18: 564–570. 10.1038/nsmb.2054 21478865

[56] Safe S, Jin UH, Morpurgo B, Abudayyeh A, Singh M, Tjalkens RB. Nuclear receptor 4A (NR4A) family—orphans no more. J. Steroid Biochem. Mol. Biol., 2015;S0960–0760: 00113–2 (In advance). 10.1016/j.jsbmb.2015.04.016 PMC4618773 25917081

[57] Zhan YY, Chen Y, Zhang Q, Zhuang JJ, Tian M, Chen HZ, et al The orphan nuclear receptor Nur77 regulates LKB1 localization and activates AMPK, Nat. Chem. Biol., 2012;8: 897–904. 10.1038/nchembio.1069 22983157

[58] Wang WJ, Wang Y, Chen HZ, Xing YZ, Li FW, Zhang Q, et al Orphan nuclear receptor TR3 acts in autophagic cell death via mitochondrial signaling pathway, Nat. Chem. Biol., 2014;10: 133–140. 10.1038/nchembio.1406 24316735

[59] Watson LC, Kuchenbecker KM, Schiller BJ, Gross JD, Pufall MA, Yamamoto KR. The glucocorticoid receptor dimer interface allosterically transmits sequence-specific DNA signals, Nat. Struct. Mol. Biol. 2013; 876–883. 10.1038/nsmb.2595 23728292 PMC3702670

[60] Osz J, Brélivet Y, Peluso-Iltis C, Cura V, Eiler S, Ruff M, et al Structural basis for a molecular allosteric control mechanism of cofactor binding to nuclear receptors. Proc. Natl. Acad. Sci. USA, 2012;109: E588–E594, 10.1073/pnas.1118192109 22355136 PMC3309777

