# Supplementary material for: In Silico Adoption of an Orphan Nuclear Receptor NR4A1
Source: PLoS One. 2015 Aug 13;10(8):e0135246. doi: 10.1371/journal.pone.0135246 (PMC4535767; doi:10.1371/journal.pone.0135246)
Supplement: S1 Text — (PDF) [file pone.0135246.s002.pdf]

S1 Text

Structure generation

The coordinates for the orphan human nuclear receptor subfamily 4 group A member 1 (NR4A1), also denoted as nuclear hormone receptor NUR77 or orphan nuclear receptor TR3, are available from the protein database as entry 2QW4.pdb.[1] The underlying sequence has the UniProt identifier P22736.[2] The structure contains 233 residues and is resolved at 2.8 Å resolution. Three amino acids (EPQ) comprising the surface-loop region of a helix-turn-helix motif are not structurally resolved. The missing amino acids were added by the loop-modeling functionality provided within the SwissModel web service available at http://swissmodel.expasy.org.[3,4,5,6]

Ligands **1**[7] and **2**[8] were constructed employing standard fragments within UCSF Chimera,[9] charged using the AM1-BCC Hamiltonian,[10] and minimized to a final gradient of 0.05 kcal $mol^{-1}Å^{-1}$ applying the GAFF force field.[11]

References and Notes

[1] Min JR, Schuetz A, Loppnau P, Weigelt J, Sundstrom M, Arrowsmith CH, Edwards AM, Bochkarev A, Plotnikov AN The RCSB Protein Data Bank. http://www.pdb.org/pdb/explore/explore.do?structureId=2QW4, Accessed 4th May 2015.

[2] The UniProt Protein Knowledgebase. http://www.uniprot.org/uniprot/P22736, Accessed 4th May 2015.

[3] Biasini M, Bienert S, Waterhouse A, Arnold K, Studer G, Schmidt T, Kiefer F, Cassarino TG, Bertoni M, Bordoli L, Schwede T (2014) SWISS-MODEL: modelling protein tertiary and quaternary structure using evolutionary information. Nucleic Acids Res 42 (W1): W252-W258.

[4] Arnold K, Bordoli L, Kopp J, Schwede T (2006) The SWISS-MODEL Workspace: A web-based environment for protein structure homology modelling. Bioinformatics 22:195-201.

[5] Kiefer F, Arnold K, Künzli M, Bordoli L, Schwede T (2009) The SWISS-MODEL Repository and associated resources. Nucleic Acids Research 37: D387-D392.

[6] Guex N, Peitsch MC, Schwede T (2009) Automated comparative protein structure modeling with SWISS-MODEL and Swiss-PdbViewer: A historical perspective. Electrophoresis 30(S1): S162-S173.

[7] Zhan Y, Du X, Chen H, Liu J, Zhao B, Huang D, Li G, Xu Q, Zhang M, Weimer BC, Chen D, Cheng Z, Zhang L, Li Q, Li S, Zheng Z, Song S, Huang Y, Ye Z, Su W, Lin SC, Shen Y, Wu Q (2008) Cytosporone B is an agonist for nuclear orphan receptor Nur77. Nat Chem Biol 4: 548-56.

[8] Lee SO, Li X, Khan S, Safe S (2011) Targeting NR4A1 (TR3) in cancer cells and tumors. Expert Opin Ther Targets 15: 195-206.

[9] Pettersen EF, Goddard TD, Huang CC, Couch GS, Greenblatt DM, Meng EC, Ferrin TE (2004) UCSF Chimera - a visualization system for exploratory research and analysis. J Comput Chem 25:1605-1612.

[10] Jakalian A, Bush BL, Jack DB, Bayly CI (2000) Fast, efficient generation of high-quality atomic charges. AM1-BCC model: I. Method. J Comput Chem 21: 132-146.

[11] Wang J, Wolf RM, Caldwell JW, Kollman PA, Case DA (2004) Development and testing of a general AMBER force field. J Comput Chem 25: 1157-1174.
