# Supplementary material for: In Silico Adoption of an Orphan Nuclear Receptor NR4A1
Source: PLoS One. 2015 Aug 13;10(8):e0135246. doi: 10.1371/journal.pone.0135246 (PMC4535767; doi:10.1371/journal.pone.0135246)
Supplement: S2 Text — (PDF) [file pone.0135246.s003.pdf]

## S2 Text

### MD simulations

All MD simulations were performed using the AMBER10 simulation package[1] with the parm99 force field modified by Hornak and Simmerling.[2,3] The protein was positioned in a periodic truncated octahedron box of TIP3P water molecules and neutralized with chloride ions using ion parameters given by Joung and Cheatham.[4] The SHAKE mode was employed for hydrogen atoms, allowing a simulation time step of 2 fs. Non-bonded interactions were treated with a cutoff of 8 Å. The simulation was performed under periodic boundary conditions at 1 atm pressure (coupling constant: 1.0 ps) and 300 K temperature with a Berendsen coupling of 1.0 ps to an external heat bath. Prior to the MD simulation, the system was minimized by 500 steps *via* steepest-descent followed by 2000 steps *via* conjugate-gradient optimization, unrestrained, reaching an RMS gradient of 0.01 kcal $mol^{-1}Å^{-1}$. During an equilibration period of 50 ns, harmonic restraints on the protein main chain atoms were reduced stepwise from 5 kcal $mol^{-1}Å^{-2}$ to zero within the first 10 ns. A total production phase of 4.1 µs was used for data collection and analysis, for which atomic coordinates, energies, and temperatures were sampled and recorded every picosecond.

### References and Notes

[1] Case DA, Darden TA, Cheatham TE III, Simmerling CL, Wang J, Duke RE, Luo R, Crowley M, Walker RC, Zhang W, Merz KM, Wang B, Hayik S, Roitberg A, Seabra G, Kolossváry I, Wong KF, Paesani F, Vanicek J, Wu X, Brozell SR, Steinbrecher T, Gohlke H, Yang L, Tan C, Mongan J, Hornak V, Cui G, Mathews DH, Seetin MG, Sagui C, Babin V, Kollman PA (2008) AMBER10. University of California, San Francisco.

[2] Hornak V, Abel R, Okur A , Strockbine B, Roitberg A, Simmerling C (2006) Comparison of multiple Amber force fields and development of improved protein backbone parameters. Proteins 65: 712-725.

[3]Wickstrom L, Okur A, Simmerling C (2009) Evaluating the Performance of the ff99SB Force Field Based on NMR Scalar Coupling Data. Biophys J 97: 853-856.

[4] Suk Joung I, Cheatham TE III (2008) Determination of Alkali and Halide Monovalent Ion Parameters for Use in Explicitly Solvated Biomolecular Simulations J Phys Chem B 112: 9020-9041.
