# Supplementary material for: In Silico Adoption of an Orphan Nuclear Receptor NR4A1
Source: PLoS One. 2015 Aug 13;10(8):e0135246. doi: 10.1371/journal.pone.0135246 (PMC4535767; doi:10.1371/journal.pone.0135246)
Supplement: S3 Text — (PDF) [file pone.0135246.s004.pdf]

S3 Text

Clustering and DASH analyses

To handle the large amount of data produced by the 4.1 µs simulation time, it was decided to reduce the data density by extracting one snapshot per nanosecond, leading to a total of 4,100 microstates for subsequent data analysis. This was achieved using the cluster analysis functionality of the PTRAJ module provided in the AMBER package. To group the configurations found in the MD simulations into distinct sets, a means algorithm based on the RMS deviation of the protein C_α-atoms was applied.[1] Snapshots representing the geometrical center of each cluster obtained were identified and served as the structural basis for subsequent simulations and analyses. A cluster analysis performed at the end of the simulation (4.1 µs) led to 15 clusters including five generated by a preliminary cluster analysis performed after 1.8 µs. The cluster centers obtained from the two analyses were subjected to cavity identification and comparison.

As an alternative to a clustering based on the RMS deviation of C_α-atoms given in Cartesian coordinates, the DASH[2] procedure was employed using the ϕ and ψ dihedral angles of the protein backbone of each residue and snapshot as data source. The analysis tool Simulaid was used to extract the dihedral angles for each snapshot from the MD trajectory.[3]

9 References and Notes

[1] Shao J, Tanner SW, Thompson N, Cheatham TE III (2007) Clustering molecular dynamics trajectories: 1. Characterizing the performance of different clustering algorithms. J Chem Theory Comput 3: 2312-2334.

[2] Salt DW, Hudson BD, Banting L, Ellis MJ, Ford MG (2005) DASH: a novel analysis method for molecular dynamics simulation data. Analysis of ligands of PPAR-gamma. J Med Chem 48: 3214-3220.

[3] Mezei M (2010) Simulaid: a simulation facilitator and analysis program. J Comput Chem 31: 2658-2668.
