# Supplementary material for: In Silico Adoption of an Orphan Nuclear Receptor NR4A1
Source: PLoS One. 2015 Aug 13;10(8):e0135246. doi: 10.1371/journal.pone.0135246 (PMC4535767; doi:10.1371/journal.pone.0135246)

S4 Text

Structures of the clusters and N-terminal loop-closing

The following overlay of snapshots extracted from the simulation after 703 ns (cyan), 1250 ns (magenta), and 4000 ns (yellow), obtained by fitting onto the starting geometry (green), clearly indicates the irreversible conformational change of the loop 175 - 185 leading from an open to a closed cavity.

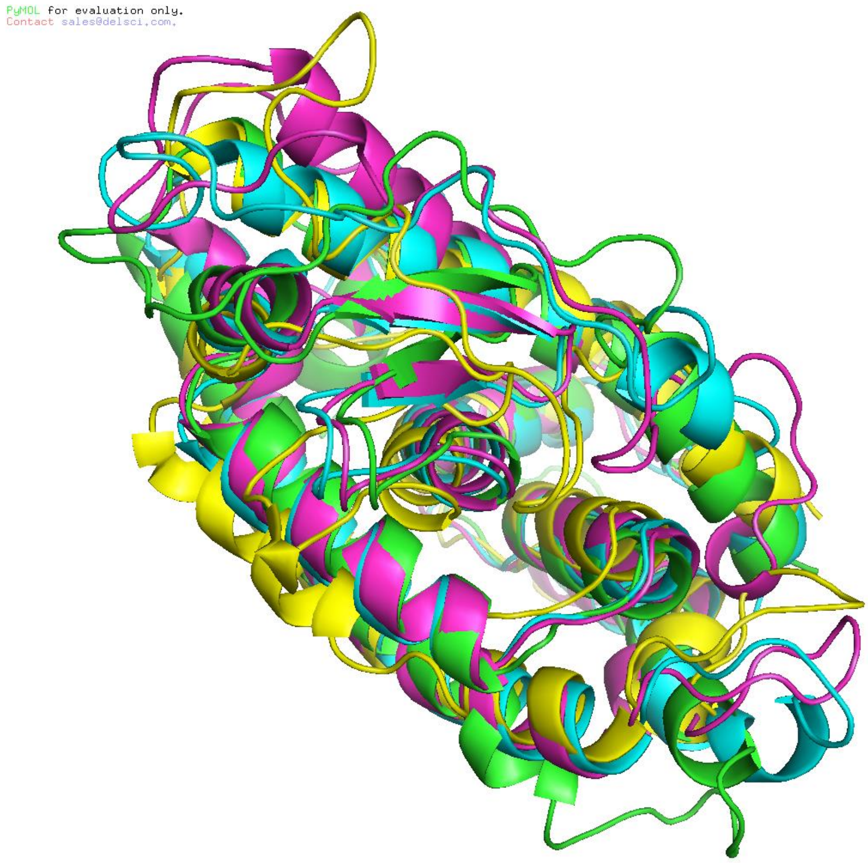

Supplement: S4 Text — (PDF) [file pone.0135246.s005.pdf]
