# Supplementary material for: In Silico Adoption of an Orphan Nuclear Receptor NR4A1
Source: PLoS One. 2015 Aug 13;10(8):e0135246. doi: 10.1371/journal.pone.0135246 (PMC4535767; doi:10.1371/journal.pone.0135246)
Supplement: S5 Text — (PDF) [file pone.0135246.s006.pdf]

S5 Text

Docking of known ligands into the new pocket

Ligands were docked into the binding site identified above using AutoDock4.[1] The protein and ligand were prepared *via* AutoDockTools 1.5.4 following standard procedures. The docking was carried out employing a Lamarckian Genetic Algorithm for searching the ligand's orientation and conformational space. The resulting docking poses were clustered by similarity of conformation and orientation. A force field that includes electrostatic interaction, a pseudo-Lennard-Jones potential, a charge-based desolvation term with hydrogen bond directionality[2,3] was used to estimate the ligand free energy of binding. By default, it was assumed that the conformation of the unbound ligand and protein are the same as those in the complex. Defining a growing number of protein side chains within the ligand-binding site as flexible does not improve the predicted binding affinity of the complex. Therefore, only the first amino acid ASN1, which is located at the border of the binding pocket, was finally treated as flexible. All docked ligands fit into the binding pocket generated by the MD simulation.

Bis-indole agonist **1**

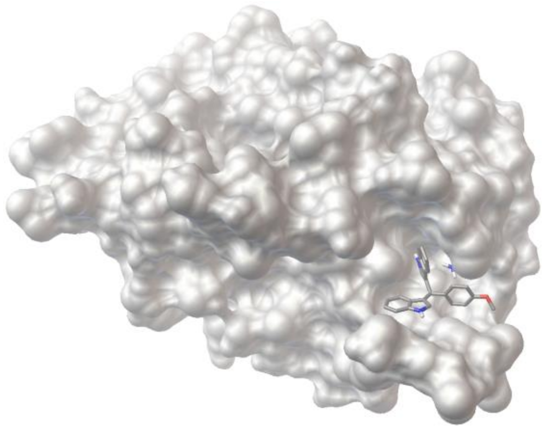

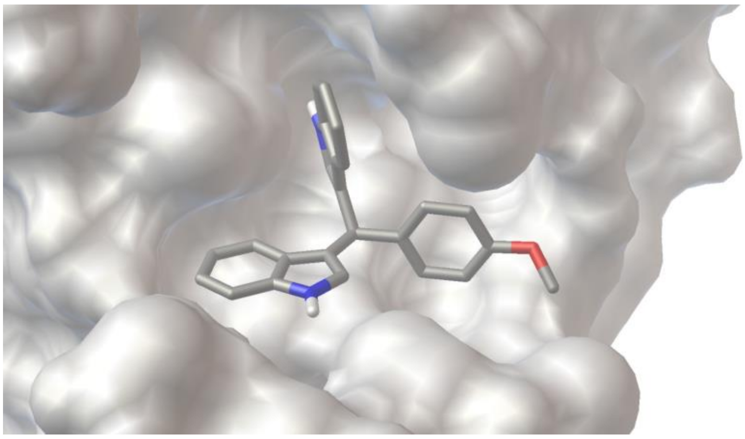

Cytosporone B agonist **2**

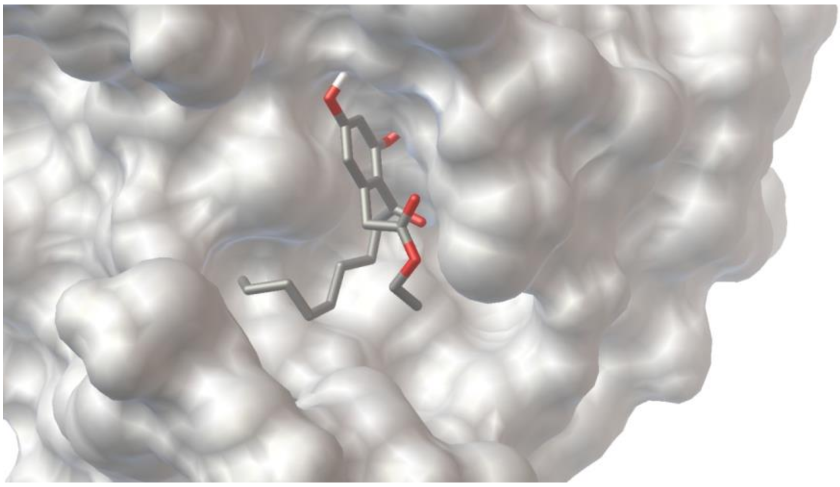

Additional ligands according to reference
Lee et al., Mol. Endocrinol. 28 (1014) 1729 – 1739.

As suggested by one of the referees, we checked a series of several p-phenyl substituted analogues of the bis-indole compound **1** (1,1-Bis(3'-indolyl)-1-(p-phenyl-OCH_3)-methane; DIM-C-pPh-OCH_3) by docking them into our new binding pocket as described above. As an overall result, all compounds fit into the pocket and show a similar binding mode and orientation. For the tested compounds, the following table gives the predicted binding affinities (in kcal/mol), according to the scoring function provided by AutoDock4:

**Compound**	**Predicted binding affinity [kcal/mol]**
DIM-C-pPh-OCH_3 (**1**)	-7.44
DIM-C-pPh-OH	-8.19

DIM-C-pPh-H	-7.77
DIM-C-pPh-I	-8.02
DIM-C-pPh-F	-7.54
DIM-C-pPh-Br	-8.08
DIM-C-pPh-Cl	-7.91
DIM-C-pPh-CH_3	-7.81
DIM-C-pPh-tBu	-8.67
DIM-C-pPh-Ph	-9.16
DIM-C-pPh-CO_2CH_3	-8.88

References and Notes

[1] Morris GM, Huey R, Lindstrom W, Sanner MF, Belew RK, Goodsell DS, Olson AJ (2009) AutoDock4 and AutoDockTools4: Automated docking with selective receptor flexibility. J Comput Chem 30: 2785-2791.

[2] Huey R, Morris GM, Olson AJ, Goodsell DS (2007) A semi-empirical free energy force field with charge-based desolvation, J Comput Chem 28: 1145-1152.

[3] Huey R, Goodsell DS, Morris GM, Olson AJ (2004) Grid-based hydrogen bond potentials with improved directionality. Lett Drug Design Discovery 1: 178-183.
