# Supplementary material for: In Silico Adoption of an Orphan Nuclear Receptor NR4A1
Source: PLoS One. 2015 Aug 13;10(8):e0135246. doi: 10.1371/journal.pone.0135246 (PMC4535767; doi:10.1371/journal.pone.0135246)
Supplement: S6 Text — (PDF) [file pone.0135246.s007.pdf]

S6 Text

RMSD data ligand 1 complex

The poses obtained by docking the putative ligands into the binding site occurred during the MD simulation of the un-complexed protein served as starting point for MD simulations of the respective complexes. Ligand **1** stays within the binding site even after 1µs simulation time illustrating an extraordinary stability for the protein-ligand complex.

RMS Cα atoms 1µs simulation with bound ligand **1**

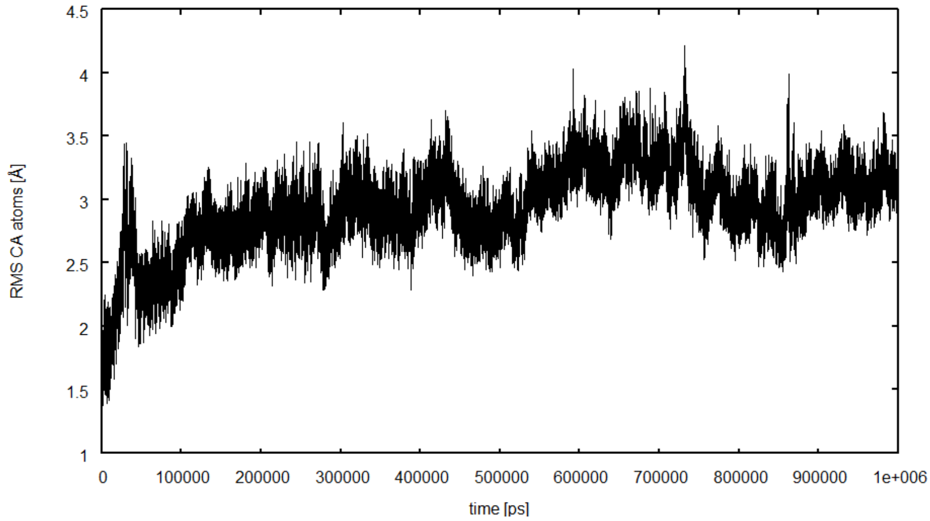

RMS ligand atoms 1µs simulation with bound ligand **1**

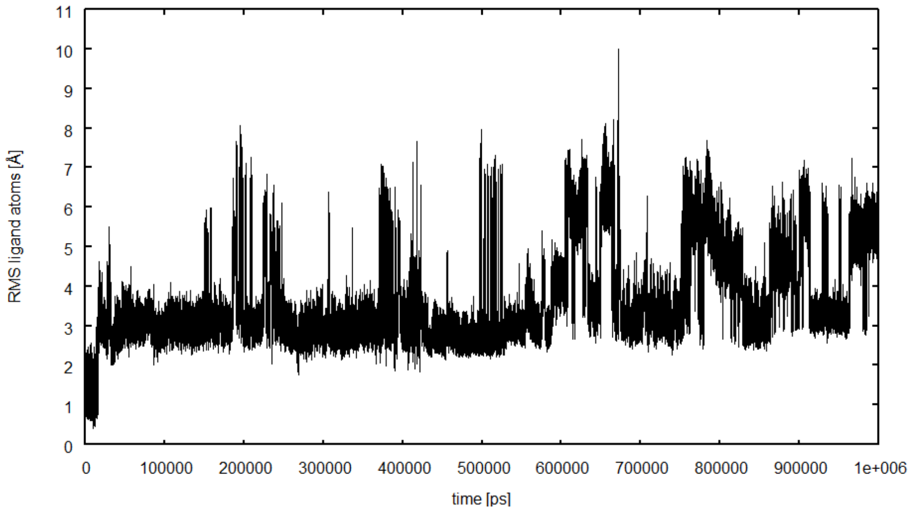

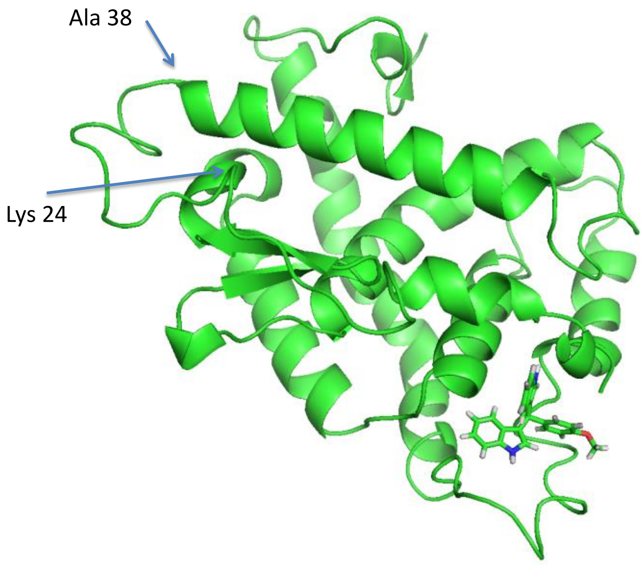

0 ns (start)

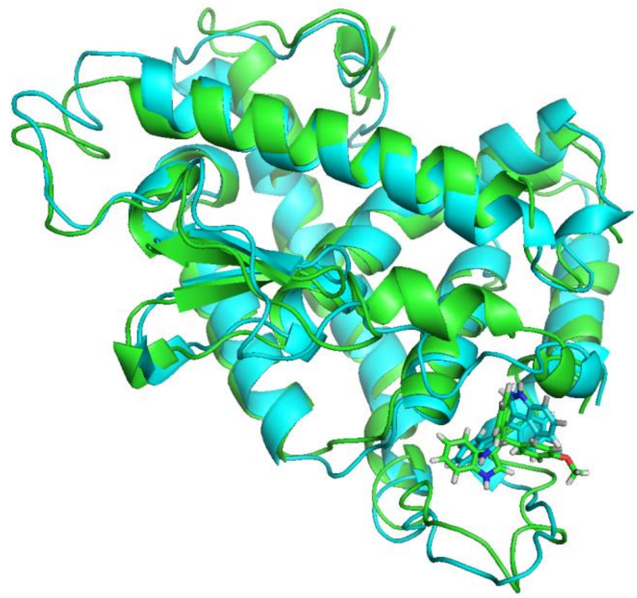

100 ns

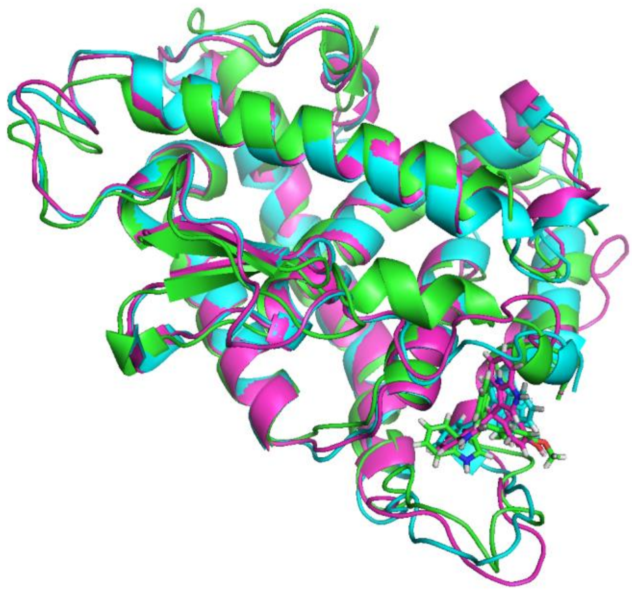

800 ns
