# Supplementary material for: In Silico Adoption of an Orphan Nuclear Receptor NR4A1
Source: PLoS One. 2015 Aug 13;10(8):e0135246. doi: 10.1371/journal.pone.0135246 (PMC4535767; doi:10.1371/journal.pone.0135246)
Supplement: S7 Text — (PDF) [file pone.0135246.s008.pdf]

S7 Text

Details of ligand 2 binding simulations

Ligand **2** agonist also stays within the binding site for the simulation time of 0.6 µs, although helix 1 covering the binding site gets kinked around the ligand. Within both simulations, the protein structure in general and the geometry of the binding cavity appears to be stabilized by the presence of the ligand.

Starting at 780 ns, the loop forming the new binding pocket shifts towards helix 1 and closes the pocket in a way that the ligand is perfectly captured. This closing is only possible because the ligand has reached a position on top of helices 8 and 9, flanked by the C-terminus of helix 2, additionally covered by the very flexible N-terminus of the protein.

RMS Ca atoms 1 µs simulation with bound ligand 2

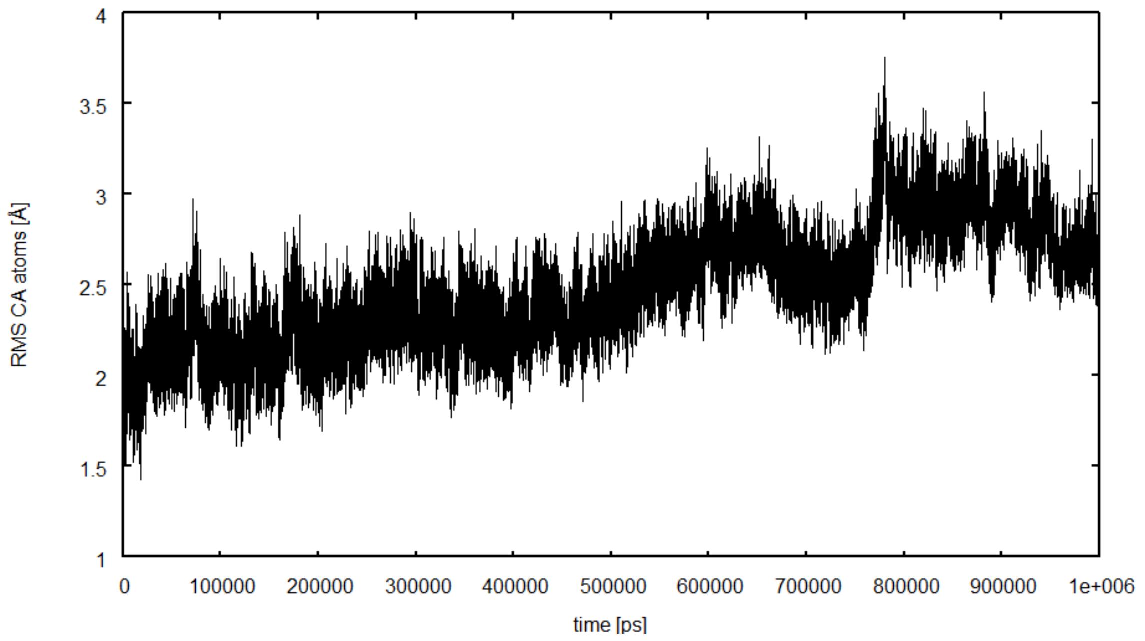

RMS ligand atoms 1 µs simulation with bound ligand 2

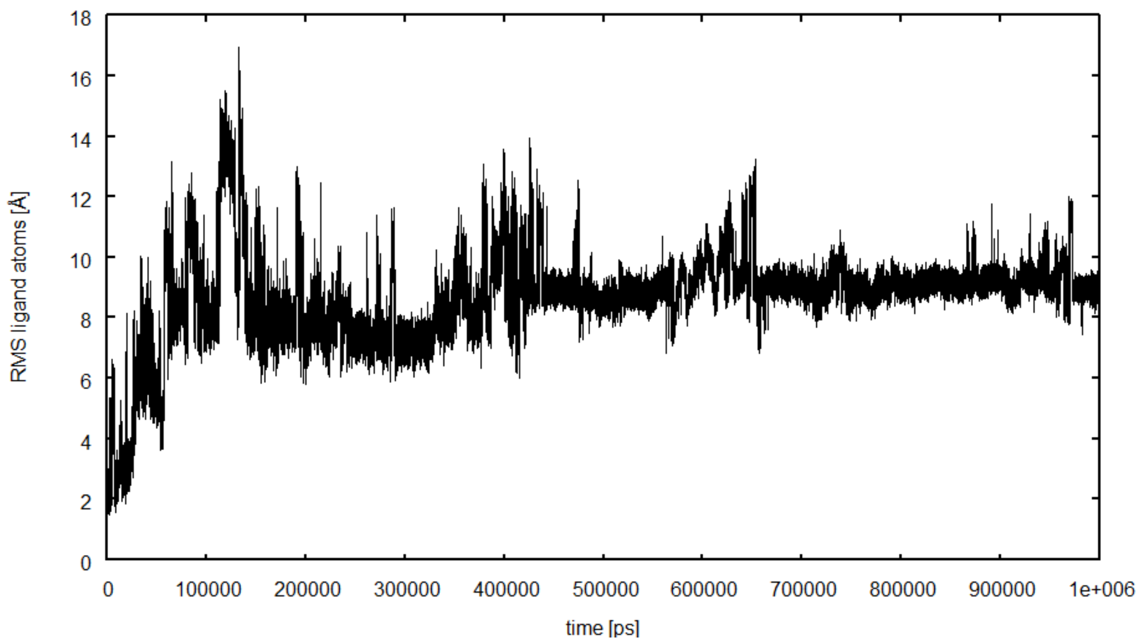

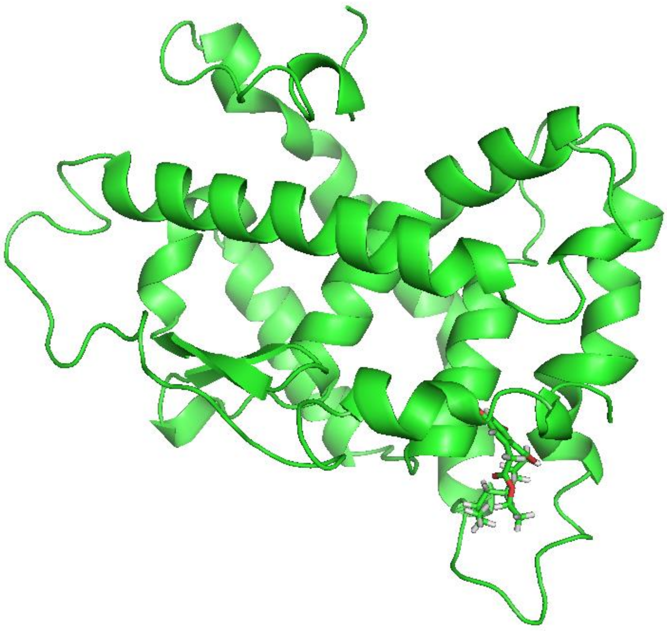

0 ns (start)

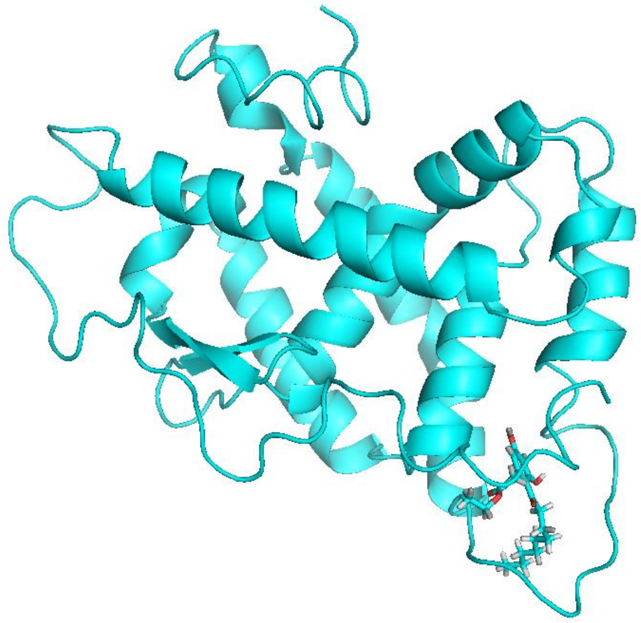

46 ns

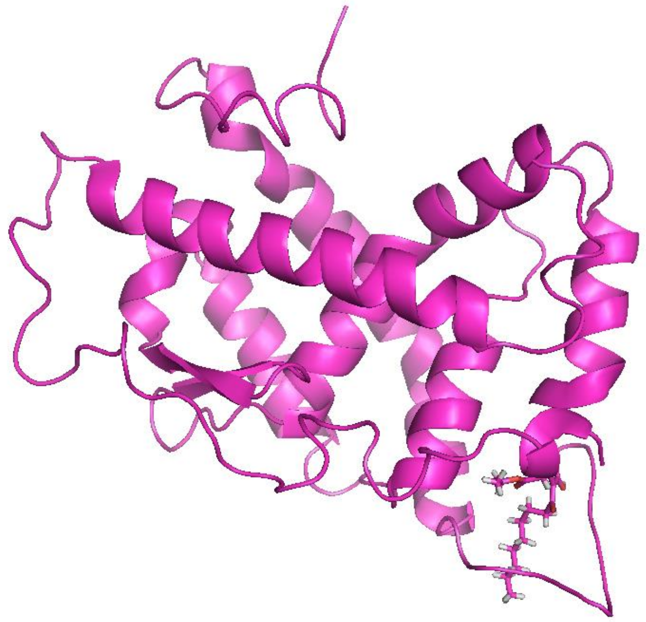

303 ns

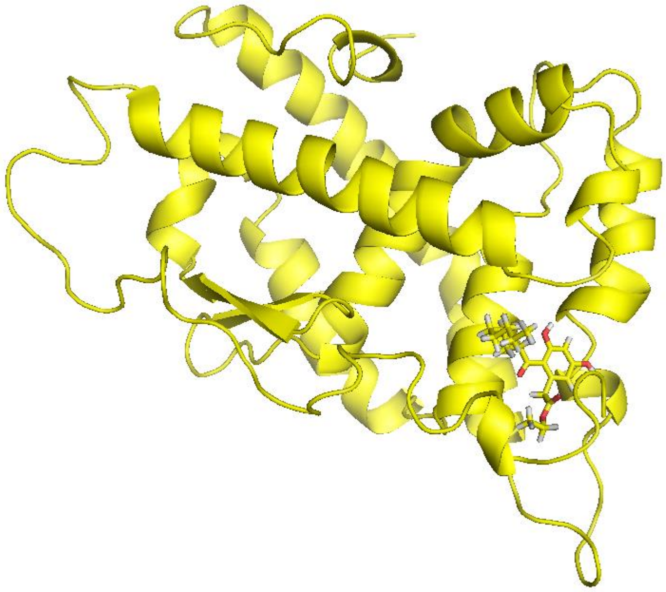

533 ns

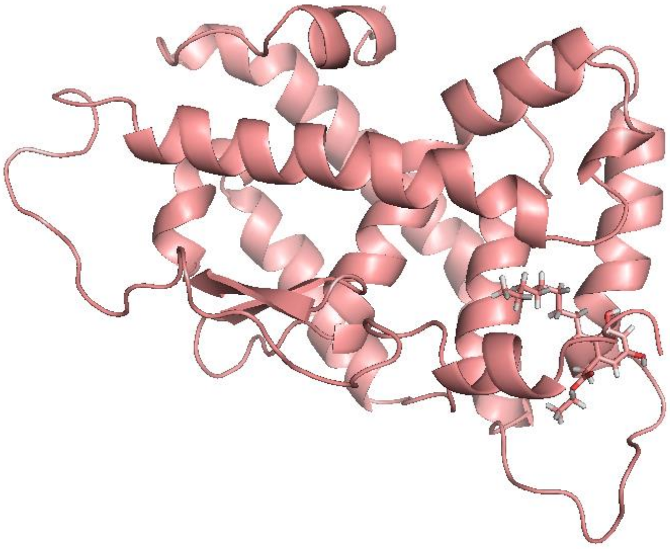

750 ns

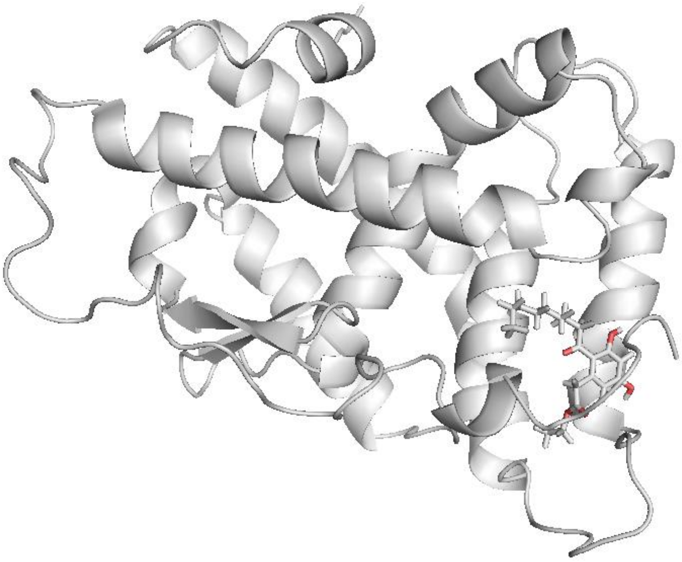

850 ns

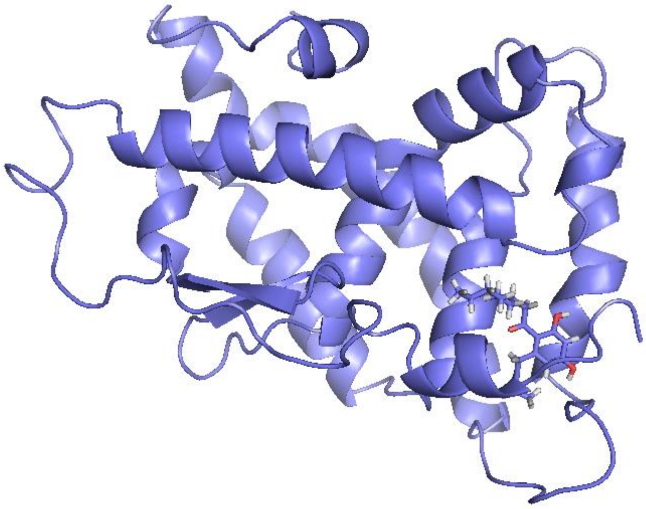

1000 ns

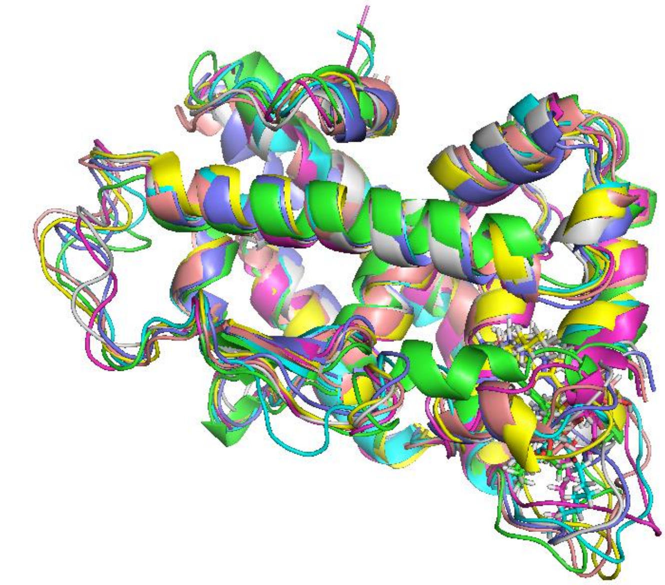

overlay
