# Supplementary material for: In Silico Adoption of an Orphan Nuclear Receptor NR4A1
Source: PLoS One. 2015 Aug 13;10(8):e0135246. doi: 10.1371/journal.pone.0135246 (PMC4535767; doi:10.1371/journal.pone.0135246)
Supplement: S8 Text — (PDF) [file pone.0135246.s009.pdf]

S8 Text

A RXR-α / hNur77 apo-dimer at a cognate NBRE

Alignment of apo-hNur77 (Coordinates from PDB:2QW4, Chain C, LBD purple cartoon cylinders) replaced the PPAR-γ monomer and was posed against the RXR-α monomer containing 9-*cis*-retinoic acid within its LBD utilizing the x-ray structural coordinates of a PPAR-γ / RXR-α / PPRE tertiary complex (PDB: 3DZY) at a recognized NuRE (Coordinates from PDB:1CIT, red and green sticks). Structural alignments were performed via the 'align' routine available within MacPymol. hNur77 with ligand **1** docked is seen as an overlay with the apo-hNur77 (brown cylinders, CPK spheres for **1**).

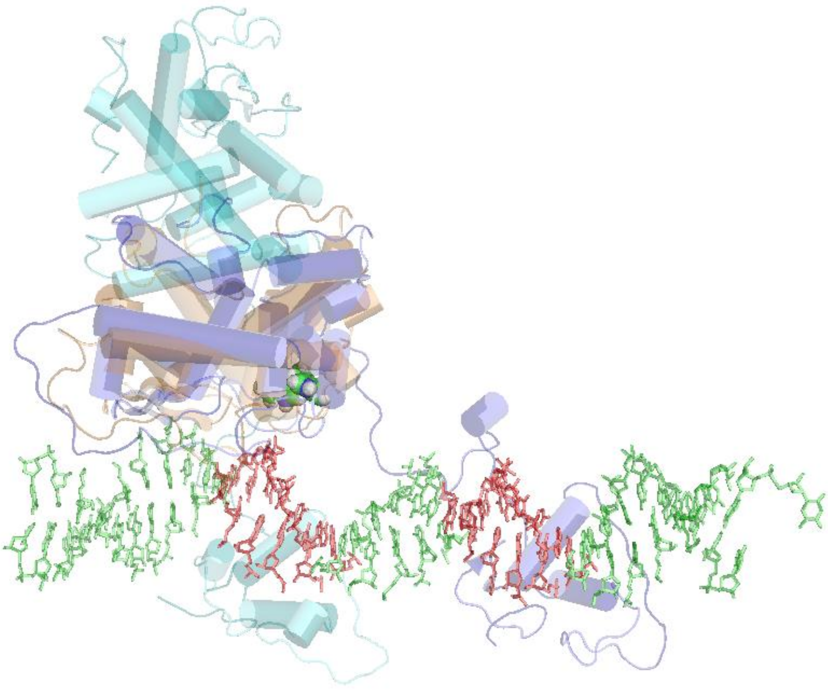

9 References and Notes
